# Women’s experiences of receiving information about and consenting or declining to participate in a randomized controlled trial involving episiotomy in vacuum-assisted delivery: a qualitative study

**DOI:** 10.1186/s13063-021-05624-8

**Published:** 2021-09-26

**Authors:** Jenny Ericson, Cecilia Anagrius, Agnes Rygaard, Lisa Guntram, Sophia Brismar Wendel, Susanne Hesselman

**Affiliations:** 1 School of Education, Health and Social Studies, Dalarna University, Falun, Sweden; 2 Center for Clinical Research Dalarna, Uppsala University, Falun, Sweden; 3 Department of Pediatrics, Falu Hospital, Falun, Sweden; 4 Department of Gynecology and Obstetrics, Falu Hospital, Falun, Sweden; 5 Department of Thematic Studies - Technology and Social Change, Linköping University, Linköping, Sweden; 6 Department of Clinical Sciences, Karolinska Institutet, Danderyd Hospital, Stockholm, Sweden; 7 Department of Women’s and Children’s Health, Uppsala University, Uppsala, Sweden

**Keywords:** Episiotomy, Experiences, Informed consent, Randomized controlled trial, Vacuum-assisted delivery

## Abstract

**Background:**

Information about and invitation to participate in a clinical trial involving an intervention during childbirth may cause fear or worry in pregnant women. The aim of this study was to describe nulliparous women’s experiences of receiving an invitation to participate in a randomized controlled trial (RCT) of lateral episiotomy versus no episiotomy in vacuum-assisted delivery (EVA trial).

**Methods:**

This qualitative study was nested in the ongoing EVA trial. Data were collected through semistructured telephone interviews with 23 women regarding their experiences of the information and invitation to participate in the EVA trial. Interviews were audio-recorded and transcribed verbatim. A qualitative content analysis was used to analyse the interview contents.

**Results:**

Three main experience categories were identified among the participants. “Timing of trial information and understanding” revealed that women preferred to obtain information about the trial early on during pregnancy. “Reasons to consent to or decline participation in the trial” encompassed a variety of reasons for women to consent, such as goodwill for science or personal benefits, or to decline, such as not wanting to be randomized or fear of increased risk of having a vacuum-assisted delivery. “Thoughts evoked regarding childbirth” were diverse, ranging from not being affected at all to having increased anxiety.

**Conclusions:**

The women’s experience of receiving an invitation to participate in an RCT of episiotomy in vacuum-assisted delivery varied widely, from immediately giving consent without further worries to increased anxiety or declining participation. Early and personal information with time for reflection was considered most satisfactory.

**Trial registration:**

ClinicalTrials.govNCT02643108. Registered on December 28, 2015. The Lateral Episiotomy or Not in Vacuum Assisted Delivery in Non-parous Women (EVA) trial was registered at www.clinicaltrials.gov.

## Background

Informed consent is a cornerstone in clinical trials and aims to respect and promote participant autonomy and to protect participants from harm [1]. Recruitment of participants to randomized controlled trials (RCTs) can be challenging due to, for example, organizational difficulties, fewer eligible patients than expected, strong treatment preferences expressed by patients and caregivers, the randomization process, or lack of clarity in the information given. This may result in slow and inadequate recruitment [2].

At least one in ten first-time mothers is reported to have an operative vaginal delivery, of whom 10–14% sustained an obstetric anal sphincter injury (OASIS), including to the anal sphincter muscles or the rectal mucosa [3]. OASIS may cause life-long anal incontinence, perineal pain, and dyspareunia and is therefore important to avoid [4]. Observational studies indicate that lateral or mediolateral episiotomy during vacuum-assisted delivery in nulliparous women reduces the risk of OASIS [5]. The EVA (Episiotomy in Vacuum-Assisted delivery) trial is an ongoing Swedish multicentre RCT that investigates whether routine lateral episiotomy compared with no episiotomy at vacuum-assisted delivery in first-time mothers reduces the risk of OASIS [6]. In the EVA trial, women are invited to participate during pregnancy and/or labour if adequate pain relief has been given and there is enough time to obtain informed consent. Consent is verified before randomization, which takes place after the decision to deliver by vacuum extraction has been made [6]. Obtaining informed consent for the EVA trial places high demands on caregivers’ information and on pregnant women to be able to receive and process information about hypothetical scenarios at the time of delivery, including vacuum-assisted delivery, perineal injury, and/or episiotomy.

Concerns have been raised that trial information on potential complications and interventions around delivery creates fear of childbirth, and research is limited with respect to how women experience the process of informed consent in trials during pregnancy and childbirth. Few RCTs involve emergency procedures during childbirth, and those that do have seldom reported the challenges of informed consent. Lawton et al. (2016), who recruited women to an RCT at the time of retained placenta, reported that recruiting and obtaining consent from women was challenging, as the eligible women could be anxious, in pain, and/or exhausted. There may also be limited time for discussion and decision-making [7]. The aim of our study was to examine nulliparous women’s experiences of receiving an invitation to participate in an RCT of lateral episiotomy versus no episiotomy in vacuum-assisted delivery.

## Method

This qualitative study collected data through telephone interviews with women who had consented to or declined participation in the ongoing EVA trial (NCT02643108) [6]. Women who were asked to participate were selected in three different ways during January–February 2019. First, women who had accepted participation during the previous 6 months could be found in the screening log for the EVA trial. Second, women who declined participation during January–February 2019 could be found from the medical record. Third, women who were still pregnant and women who declined or accepted participation could be found from midwives at maternity health care centres. In total, 26 women were approached. Of these, 24 women gave consent to participate in our study, and two declined. Efforts were made to recruit a purposive group of women to achieve a diversity of experiences about being informed and consenting or declining to participate in the EVA trial. For example, the women who participated lived in either a rural or a metropolitan region in Sweden. The background characteristics of the participating women are shown in Table 1. Twenty-two women had given birth at the time of the telephone interview, and one was still pregnant. Most participants were of Swedish origin, cohabiting with the other parent, and employed or self-employed. The Regional Ethics Review Board in Stockholm approved the study (Dnr 2015/1238-31/2 with amendments 2018/775-32 and 2018/2291-32).

**Table 1:** Background characteristics of the participating women (*n* = 23)

	*n* (%) Mean [range]
Age (years)	30.6 [23–38]
Country of birth	
*Sweden*	20 (87)
*Outside Sweden*	3 (13)
Family situation	
*Cohabitant with partner*	22 (96)
*Single mother*	1 (4)
Place of residence	
*Stockholm region*	2 (9)
*County of Dalarna*	20 (87)
*Others*	1 (4)
Occupation	
*Employed/self-employed*	19 (83)
*Social benefits/student*	4 (17)
Consent to EVA trial	
*Yes*	14 (61)
*No*	9 (39)
Mode of delivery	
*Spontaneous vaginal*	15 (66)
*Vacuum extraction*	4 (17)
*Caesarean section*	4 (17)

The author CA scheduled and performed all telephone interviews in February through April 2019. A semistructured interview guide was used, encompassing three main themes: information given and received, the process of consent, and concerns and fears. The data collection was stopped after the 24th interview because similar content recurred (data saturation was reached). Twenty-three telephone interviews were audio-recorded on tape and transcribed verbatim. In one interview, the recording did not work, and this interview was excluded from the analysis. The interviews lasted 4 to 10 min.

### Data analysis

Qualitative content analysis inspired by the work of Elo and Kyngäs was used on the transcribed text from the 23 interviews [8]. Each of the authors JE, SH, AR, and CA read through the transcribed texts several times to familiarize with the content. Thereafter, open coding was applied to the transcriptions, whereby the content was summarized. The codes were grouped into subcategories based on similarities and patterns. The subcategories’ similarities and patterns were discussed and abstracted to main categories by the authors. The main categories served to identify the women’s experiences of being informed about and asked to participate in the EVA trial. Each category was named using words that were characteristic of its content. The wording of the extracted data was kept as similar as possible to the women’s original statements during the entire analysis to maintain the transparency and trustworthiness of the outcome [9]. Interviews were called I 1 to I 23, and quotes from the interviews are included in this text. All identifying data were removed from quotes used in this text. Table 2 presents examples of the analysis process.

**Table 2:** Examples of the analysis process of the 23 transcribed telephone interviews regarding receiving an invitation to the randomized controlled trial

Interview	Original statement	Subcategory	Main category
16	Mm, they asked when I got in there [labour ward], if I wanted to participate in this study, and I had heard about it before also at the midwife, that this study existed.	Where did the woman get information?	Timing of trial information and understanding
7	I think it is always important to contribute to everything like that [the RCT], which can improve care and research and everything like that. Because I know it is necessary. I said yes to participate pretty quickly, but then they said: “Yes, but you don’t have to decide now, you have to think.” “Yes, but I don’t have to think about it, it’s okay, I can be part of it.”	Wants to contribute to research	Reasons to consent or decline
9	I might not have conceived of such a thing, with a vacuum-assisted delivery. I probably didn’t even want to think about it, so that was probably when I got [a feeling] – yeah, that could happen.	Complications became actual and real	Thoughts evoked regarding childbirth

## Results

Women’s experiences were categorized into three main categories: “Timing of trial information and understanding”, “Reasons to consent or decline”, and “Thoughts evoked regarding childbirth”.

### Timing of trial information and understanding

The women had different opinions regarding whether they received information about the RCT at the right time. Most women who were informed and asked for consent in the second trimester thought it was good timing. However, some women who received information towards the end of pregnancy thought it was too late. They wanted to have the opportunity to ask questions but felt the time was too short for that, for example, in postdate pregnancy.

Well, I got it (the information) rather late. I was already beyond term when I met my midwife and got the folder, by then I was already almost, well, one and a half week beyond term, and I might have wanted it [the information] a bit earlier. (I 9)

It would have been better for me to be asked earlier in pregnancy and get the opportunity to ask questions at the next visit. (I 4)

The women who received information and were asked for consent after the onset of labour generally thought that it would have been better to receive the information during pregnancy. Some women specifically mentioned that they were tired or unprepared, did not have time to think, or were mentally affected by labour and therefore felt vulnerable and did not think it was a good time to ask for consent. One woman even said that she was not in full possession of her senses when she consented, as she was very tired, hungry, and had severe pains.

They came in the morning, before she arrived, and asked, by then we had been there for over 12 hours. And it might not have been the best way for me, but I guess it was a little different for me, since all my antenatal care had been [in another town] and they don’t have the EVA trial there, so I didn’t know it existed. Then I got some folder that I was supposed to read through and both me and my partner were pretty tired. We tried to read through as best we could. I think it would have been easier if we had been prepared before we came there, so to speak. (I 2)

Many women received information at their maternity health care centre. They received written and verbal information from their midwife and could ask questions and obtain answers. Some women received written information in a letter sent to them, and some had received verbal information at antenatal courses. The women stated that they understood the information and what the RCT was about.

Yes, absolutely, I understood it [the information]. But I wonder, either I didn’t understand, or I don’t remember now, if one was informed what the gold standard was if you didn’t participate in the study. It might have been written out, but I don’t remember it now. But I clearly understood what the purpose of the trial was. (I 4)

Some women described problems regarding the informed consent process. The women had to read the information themselves, no one followed up on the information, or they did not receive information before labour. One woman described that the midwife did not have enough knowledge about the RCT and that the attending midwife student explained it better. One woman said that she did not understand the RCT. Another woman, who had received written information earlier, said that she appreciated the verbal information given at the delivery ward.

… but I thought it was better to talk to a midwife or whatever she was. Talking to her, so you got it more verbally. I wasn’t really fit to read then, right then, like reading a brochure. (I 1)

### Reasons to decline or consent to participation

The second main category was reasons to decline or consent to participation. The women’s reasons for consenting to participate in the RCT were to contribute to science, to determine what the best treatment would be, and to prevent suffering for others. They thought it was an obvious choice and important to participate. Another reason to participate was to obtain personal benefits from the RCT. The women found the follow-up programme positive and attractive. Some women consented because they felt that participation was neutral; there was no difference between participating and not participating, as it was just as much a lottery whether they would receive an episiotomy or not outside the trial. Alternatively, they said that they believed that a vacuum-assisted delivery was very unlikely and that they might just as well participate.

Well, me and my partner went through, or discussed, what we should do. We agreed that it was as much a lottery not to participate, depending on which doctor you get, that you might as well be a part of it […] what really was a pro was the amount of check-ups you would get after the delivery. Like, several years afterwards. That you don’t ordinarily get. That was a good thing to me. (I 3)

Women who declined participation stated that they wanted to decide about episiotomy themselves. Several women said that they did not want to be randomized. Some women had read on the Internet that delivery was better with episiotomy and wanted that if they should require a vacuum-assisted delivery. They did not want a spontaneous perineal injury. Trust or lack of trust in the health care professionals were other reasons to decline or consent to participation. Some women, both those who declined participation and those who consented, trusted the staff to do the best thing. Conversely, some women described a fear that participation in the RCT would increase the risk of vacuum extraction and therefore decrease participation.

At a subconscious level I imagine that if you participate in the trial, the person who decides might subconsciously be a bit more inclined to do a vacuum. It’s just one of those spontaneous fears that exists but I’m sure isn’t valid. (I 4)

Others were simply determined not to participate in a trial or felt that they were so close to giving birth that they had not had a chance to make up their mind, were in too much pain, or were nervous and therefore did not want to participate.

But when they asked me at the delivery ward when I arrived there, by then I was awfully preoccupied by pain, and I was so damn nervous about the whole delivery and everything, that I felt I couldn’t focus on another thing to be nervous about. I sort of wanted to trust that the doctor would do what was best in that moment in case it came to a vacuum. Because I didn’t want to be in the lottery, like in this situation. (I 12)

### Thoughts evoked regarding childbirth

Various thoughts regarding childbirth were evoked when being informed about and invited to participate in the EVA trial. Some women were not affected at all by the information and invitation, nor did it result in any added worries or thoughts about childbirth.

I really didn’t have that many thoughts, I thought that I’ll take it as it comes and what will be, will be. I can’t do anything about it anyway. (I 17)

Just another thing to decide on. But not more worry. I wouldn’t say that. (I 3)

Information about and invitation to participate in the trial raised worries in some women. These women said that childbirth became actual and real. It seemed unpleasant and frightening with a vacuum-assisted delivery, a perineal or vaginal injury, or an episiotomy. Some women stated that consenting to participate in the trial was a simple choice during pregnancy but that other feelings emerged during childbirth and could be very distressing. One woman who consented to participate said that when the staff started talking about a possible vacuum-assisted delivery, she felt “ugh, can I escape?” (I 5). Another woman who had consented to participate described her feelings during childbirth as follows:

First, I did not think that far, just – well of course I’ll participate, and so I said yes, and I didn’t give it much thought, until we came to the part when she had to come out. Because then, at some subconscious level I started like ‘what if I end up in the cut group and they cut although they shouldn’t, or what if I end up in the tear group and they don’t cut even though they should?’ […] So all that time I just focused on ‘am I tearing, am I tearing? I don’t want you to do the vacuum! I don’t want you to do the vacuum! Please, what’s happening? You have to tell me!’ I was really worried in that moment that it would have negative consequences in the course of events. Which I really wasn’t prepared for! (I 7)

Overall, there was a wish to avoid vacuum extraction and for a normal delivery without complications. Several women expressed fear of sustaining a perineal or vaginal injury, and they thought it was better to receive an episiotomy. There was also a lack of knowledge among some women about vacuum-assisted deliveries. In some cases, stories from friends and family members about problems after tearing and vacuum-assisted delivery affected the women.

It [the thought of the delivery] is very much coloured by the stories you have been told by friends and family and so on. Because, well, I have had a hard time imagining my own delivery in any way. Anything can happen, and I have to face that somehow. (I 18)

## Discussion

This study describes women’s experiences of the process of giving or withholding informed consent to participate in an RCT of lateral episiotomy versus no episiotomy during vacuum-assisted delivery. There was a wide range of experiences regarding the timing and content of the trial information provided. The women expressed more satisfaction when information was given early on during pregnancy and stated that they were more receptive before the onset of labour. They felt less satisfaction when consent was requested during labour, when they were affected by pain, fatigue, or anxiety. Experiences ranged from not being worried by the trial information at all and giving immediate consent to becoming more anxious about childbirth or declining participation.

The observed variation in experiences regarding receiving and understanding the trial information may reflect the differences in approaches among the recruiting clinical staff, such as knowledge and views on evidence, RCT design, role conflicts, and personal preferences [10]. To avoid a difficult informed consent process depending on clinical staff, other options would be an open trial [11], an “opt-out” instead of “opt-in” approach [12], cluster randomization (without consent) [13], or patient preference designs [14]. However, all these strategies and methods are prone to methodological and ethical challenges. Studies on how to improve recruitment strategies in RCTs are scarce, and patient involvement in the development of information folders has a limited effect on participation rates [11]. Therefore, qualitative methods, such as our study, may expose potential pitfalls, which in turn may be used as a basis for new strategies for improved recruitment [10].

First, our study highlights the dilemma that can arise when recruiting women during pregnancy or labour to an interventional trial of a hypothetical emergency procedure. What felt like an easy decision during pregnancy later became a distressing experience with a wish to opt out. This illustrates the difficulty of comprehending early information about an intervention in relation to how one will feel later, in the moment when it is actually going to take place. Second, the information given may be inadequate for situations arising later. On the other hand, late information may hamper informed consent. Like our study participants, women in a trial regarding retained placenta raised retrospective concerns about whether they had actually been able to make a fully informed decision, although their informed consent was given freely at the time of consent [7].

Third, modern individualized care should involve shared decision-making and the possibility of influencing care given during childbirth [15]. Nevertheless, the decision to perform an episiotomy is rarely shared by the woman, which is often justified by the medical urgency and perceived incapacity of the woman to make an informed decision at the time of episiotomy [16–20]. In emergency medicine research, waiving informed consent is common when the research subject is (temporarily) incapacitated from giving informed consent, the treatment window is short, or prospective informed consent is not possible, as potential participants are impossible to identify prospectively [21, 22]. This could also apply to interventional trials during childbirth, such as the EVA trial. However, childbirth is not an unexpected event during pregnancy, and some argue that women do not typically lose the ability to make decisions during childbirth [23], although some women in our study expressed such feelings. A woman giving birth is in a vulnerable position, where she must rely on health care staff but is usually more actively involved in her care than an ill patient. We hypothesize that the feeling of lost decision-making ability is more tangible during childbirth than during illness. Thus, efforts to give thorough and timely information before any urgent situation occurs seem essential to maintain a sense of autonomy.

However, our study also showed that several women thought that the research was important and that the choice to participate was obvious. This may be due to plentiful information on perineal injuries in the media and frequent benchmarking between labour wards in the effort to decrease the rates of OASIS [24]. Knowledge of the research field and previous experience from research have been found to be important factors influencing women’s willingness to take part in a clinical trial during pregnancy [25]. It should also be acknowledged that the women in our study expressed concerns about being viewed and treated as research objects and not as individuals. Some women in our study were repelled by the idea of random assignment and wanted to get the treatment preferred by themselves or their caregiver [26], although randomization is essential in an RCT and the rationale of an RCT is to find the best practice. This illustrates the complexity of giving and receiving adequate information to facilitate decision-making. Efforts should be made to explain randomization, including the physician’s option to deviate from the allocated treatment if deemed necessary and the patient’s right to withdraw consent at any time. This creates high demands on recruiters with respect to their ability to answer questions and provide comprehensible information. To ensure such competence among recruiters, researchers must explain the aim, rationale, and design of the study in a coherent manner.

### Strengths and limitations

Strengths in our study included the approaches used to attain scientific rigour and trustworthiness. First, we aimed to achieve a broad understanding of different experiences of the informed consent process. Second, the data collection during the telephone interviews continued until similar content recurred to ensure data saturation. Third, during the analysis process, we closely retained the original statements from women to strengthen the credibility and confirmability of the findings. These measures help make our results transferable to similar populations and useful when planning the informed consent process in clinical trials. A limitation in our study is that the relatively short and telephone-based interviews may have prevented more detailed descriptions of experiences. Furthermore, the women were interviewed weeks to months after the informed consent process, which could result in less precise memories influenced by the childbirth experience. The majority of approached women had Swedish as their native language, which may limit generalizability.

## Conclusion

The women’s experiences of receiving an invitation to participate in an RCT of lateral episiotomy versus no episiotomy during vacuum-assisted delivery varied widely, from immediate consent without further worries to increased anxiety or declined participation. Women expressed more satisfaction when information was given early on during pregnancy before the onset of labour and when there was enough time to ask questions. Women appreciated personalized information explaining randomization to reduce the feeling of being a research object. Consent may change, and the right to withdraw consent should be explicit. Further research should aim to identify specific measures to improve the process of informed consent when designing and conducting trials during childbirth.

## Data Availability

The datasets generated and analysed during the current study are not publicly available for ethical and legal reasons.
